# Locus of control and anti-hypertensive medication adherence in Ghana

**DOI:** 10.11694/pamj.supp.2014.17.1.3433

**Published:** 2014-01-18

**Authors:** Irene Akwo Kretchy, Frances Thelma Owusu-Daaku, Samuel Danquah

**Affiliations:** 1 Department of Clinical and Social Pharmacy, Faculty of Pharmacy and Pharmaceutical Sciences, Kwame Nkrumah University of Science and Technology, Ghana; 2 Department of Pharmacy Practice and Clinical Pharmacy, University of Ghana School of Pharmacy, College of Health Sciences, Ghana; 3 Department of Psychology, University of Ghana, Legon, Ghana

**Keywords:** Locus of control, personality, bi-local expectancy, medication side effects, medication non-adherence, hypertension, individualism, collectivism, culture, Ghana

## Abstract

**Introduction:**

Medication non-adherence is a major public health problem in Ghana. Locus of control (LoC) may influence adherence to medication. In this study we examine the association between locus orientation and adherence to hypertensive medication among adult patients. We also take into account the role of medication side effects.

**Methods:**

We conducted a hospital-based cross-sectional study involving two tertiary hospitals in southern and northern Ghana. Data were collected from 400 hypertensive patients using a structured questionnaire. We gathered information on patient’s sociodemographic characteristics, health LoC, side effects of anti-hypertensive medication and adherence to anti-hypertensive medication.

**Results:**

Participants exhibited features of mixed LoC (both internal and external) usually referred to as bi-local expectancy. However, orientation was skewed towards external LoC. Females were marginally more likely than males to have an internal LoC. Education was associated with a greater likelihood of internal LoC. While most patients (93.3%) poorly adhered to antihypertensive medications, logistic regression model revealed that non-adherence was significantly associated with low internal LoC, medication side effects and the combined effect of medication side effects and external LoC.

**Conclusion:**

Medication non-adherence, experiences of medication side effects and LoC are associated. Multifaceted intervention programmes highlighting personality characteristics like LoC may improve anti-hypertensive medication adherence.

## Introduction

As the global disease burden shifts from communicable to non-communicable diseases, hypertension has become a key public health problem. Hypertension is the leading risk factor for cardiovascular diseases and is a major cause of death globally [1]. Hypertension affects approximately 11% - 42% of Africans [2–5]. Twagirumukiza and colleagues [3] have projected that by 2025, 125.5 million people in sub-Saharan Africa will be affected. The prevalence of hypertension in Ghana has been estimated at 25% in urban and 20% in rural populations [6].

Hypertension requires long term management and follow-up. Adherence to therapy is a key component of management. Both pharmacologic (medications) and non-pharmacologic (psychotherapy, lifestyle) therapies are prescribed for hypertensive patients with the expectation that patients will be adherent [7–9]. However, many patients fail to adhere to treatment recommendations resulting in less than optimal treatment [10, 11]. The WHO estimates that adherence rate to pharmacotherapy for hypertension is 50% [12]. According to Buabeng and colleagues [13], the level of non-adherence to anti-hypertensive medication in Ghana is about 93%. Medication side effects are often cited as the main barrier to adherence [10–12, 14–16].

Personality constructs like the locus of control (LoC) can influence health behavior and impact on illness and treatment [17]. The LoC concept stems from Rotter’s social learning theory [18] which posits that individuals can be differentiated in terms of their internal or external source of control. People with an internal LoC take responsibility and decisions without any form of influence from the external world. Studies have shown that internally-driven people are more likely to adhere to prescribed treatment regimens because they believe in their ability to influence their health [19, 20]. Conversely, individuals with external LoC assign their experiences to forces in the outer world such as chance, fate or other people. Externally-driven people are thought to be less likely to adhere to therapy because of the belief that their actions may not appreciably affect health outcomes [21, 22]. Studies have shown that internal LoC is associated with adherence to daily peak expiratory flow rate monitoring recommended for asthma management [19, 21], maintaining a gluten-free diet in the control of celiac disease [23], diabetic management [24], and use of antipsychotic medications in the management of schizophrenia [22].

Some studies have demonstrated that individuals are not entirely internal or external in their LoC; rather they consider different sources of control [18]. This leads to an exhibition of a mixed LoC also referred to as “bilocal” LoC [3] or “responsible internal” [25]. In addition, although LoC is often thought to be stable particularly in adulthood [26, 27], there is evidence suggesting that LoC may change over time [28]. Internal health LoC is associated with knowledge and attitude, psychological state, health behaviour, and better health conditions. External health LoC, on the other hand, is linked with negative health behaviours and weak psychological state [29]. High internal LoC has been found to be beneficial when barriers to medication adherence (e.g., side-effects, forgetting to take medication, and keeping track of pills) are low.

Although differences in locus orientation have been shown to be associated with health behaviour, differences in adherence behaviour in relation to LoC may be due to differences in the cultures of particular groups of people. Culture often drives societal values and distinguishes different groups of people [30]. Contrary to many studies conducted in the West that have found an association between a high internal LoC and positive health behaviours, studies conducted in other parts of the world have found different associations. Azlin and colleagues [31] observed, for example, that Malaysians with high internal LoC reported poor adherence to medications. This observation underscores cultural variability in locus orientation. Unlike western cultures which are more individualistic encouraging internal LoC, Eastern cultures tend to value collectivism, thus promoting external LoC [32, 33]. In individualistic cultures, individuals have greater personal autonomy and value the pursuance of individual goals of health and well-being to a large extent. In collectivist cultures however, individuals may not feel personally responsible for their health seeking behaviours due to greater social influence from the external world. Thus, an individual with an internal LoC in an individualistic culture may take great responsibility for his or her successes and failures about health whereas an individual with an internal LoC in a collectivist culture may take responsibility for successes and failures while placing emphasis on external factors. Although in South Africa one study showed a preponderance of bi-local expectancies [34], information regarding having this mixed Loc in view of the African cultural belief and medication adherence is inadequate. Additionally, cultural orientations may affect the responses of hypertensive patients towards their experiences with side effects. In general, however, there is a paucity of information regarding LoC, side effects and hypertensive medication adherence among Africans [20].

This study sought to examine the association between LoC and adherence to medications among hypertensive patients in Ghana. In addition, we assessed the possible influence of LoC on the association between medication side effects and adherence behaviour. We postulated that hypertensive patients with high internal LoC would be more adherent than those with external LoC. We also hypothesized that medication side effects would be associated with non-adherence, but that the association will be moderated by LoC.

## Methods

### Study setting

This cross-sectional study was conducted in two tertiary hospitals in southern and northern Ghana between May and July 2012. Korle-Bu Teaching Hospital (KBTH) is the premier and largest teaching hospital. KBTH serves the Greater Accra Region and surrounding areas. Komfo Anokye Teaching Hospital (KATH) is located in Kumasi, the Regional Capital of Ashanti Region. KATH is the second-largest hospital in the country. The hospital serves as the main referral facility for the Ashanti, Brong Ahafo, Northern, Upper East and Upper West Regions. Ashanti and Greater Accra are predominantly urban and are estimated to be home to over one-third of Ghanaians [35]. Both hospitals serve patients from diverse socio-cultural backgrounds.

### Study population

Study participants consisted of 400 hypertensive patients attending KBTH (n=200) and KATH (n=200). The minimum total sample size was determined using the statistical formula below where N is the required sample size, Z is the critical value corresponding to a 95% confidence interval (1.96), P is the estimated prevalence of hypertension (28.7%), and d is the desired level of precision taken here as 0.05 [36]:

N = Z2 P (1-P) = 315/d2

Par¬ticipants were selected through a simple random process using a random number table [37]. Participants were adult male and female patients (above 18 years), diagnosed with hypertension or who were co-morbid with other health conditions and reported having a prescription for at least one anti-hypertensive medication. Newly diagnosed, or patients who were not on any form of prescribed anti-hypertensive medications as well as in-patients, pregnant women and the incapacitated were excluded from this study.

### Study instrument

Data were collected using a researcher-administered semi-structured interview. Each interview lasted about 30 minutes. Measures included socio-demographic characteristics, health locus of control, adherence to medication, and side effects experienced. Health locus of control was assessed using the Multidimensional Health Locus of Control (MHLC) form C [38]. The MHLC scale measures variations in LoC beliefs in relation to health conditions [39] and assesses the extent to which a person believes that health status is influenced by one’s actions, ‘chance’ and ‘powerful others’ (specifically ‘doctors’ and ‘others’) [40]. The MHLC Form C included 18 items assessing whether the respondents agreed or disagreed with belief statements about hypertension (Cronbach’s alpha= 0.90).

Medication adherence was measuring using the Morisky Medication Adherence Scale (MMAS) [41] which comprises 8 items (Cronbach’s alpha = 0.79). MMAS scores can range from zero to eight with higher scores reflecting higher adherence. Participants were categorized as having low adherence, medium adherence and high adherence based on the number of positive responses obtained. Patients who scored low and moderate were eventually grouped in one category for analysis purposes [42].

The frequency at which patients experience various side effects associated with different anti-hypertensive medications was assessed with the 18-item Hypertensive Medication Side Effect Experience Scale (HMSEES) (Cronbach’s alpha = 0.80). Responses were scored on a 5-point frequency response scale that ranged from never (0), rarely (1), sometimes (2), very often (3), and always (4). Item scores were added and responses categorized as low (score of 0-24), moderate (25-48) and high (49-72) experience of side effects.

### Data analysis

Data were analyzed using the Statistical Package for Social Sciences (SPSS) version 20 and R version 2.15.2. Descriptive statistics were computed to summarize the characteristics of study participants. The chi-square test was used to assess the bivariate association between health LoC and socio-demographic characteristics. Logistic regression analysis was used to assess the association between health LoC, side effects and medication adherence while adjusting for socio-demographic characteristics.

### Ethical issues

The Institutional Review Boards at the Noguchi Memorial Institute for Medical Research, Accra, and Committee of Human Research, Publications and Ethics, Kumasi approved all procedures used in this study. All participants provided informed consent from prior to data collection.

## Results

Four hundred hypertensive patients participated in the study (Table 1). The majority of study participants were 50- 59 years old, female (62.7%), and married (63.5%). Fifty-four percent had attained a minimum of secondary school education and 89.5% were Christians. Eighty-three percent of participants resided in urban communities. Eighty percent had been diagnosed with hypertension for 10 years or less.

**Table 1:** Descriptive characteristics of participants (N=400)

Variable	Percentage
**Sex (%)**	
Male	37.3
Female	62.7
**Age (%)**	
< 20	0.3
20 – 29	3.0
30 – 39	5.0
40 – 49	17.8
50 – 59	32.5
60 – 69	26.3
≥ 70	15.3
**Marital status (%)**	
Single	9.8
Married	63.5
Widowed	18.3
Divorced/ Separated	6.3
Co-habiting	2.3
**Education Level (%)**	
No formal	12.0
Primary	8.3
Secondary	54.3
Tertiary	25.5
**Religious affiliation (%)**	
Christian (spiritual)	4.5
Christian (Charismatic/Pentecostal) Christian	41.5
(Orthodox)	43.5
Muslim	5.0
African Traditional Religion	1.0
Other	4.5
**LoC (Mean/ standard deviation)**	
Internal LoC	1.54/ 0.499
External LoC	1.50/ 0.501
**Experience of medication side effects (%)**	
Low	39.8
Moderate	53.0
High	7.2
**Medication adherence (%)**	
Poor	93.3
High	6.7

Variations in internal and external LoC were minimal indicating a mixed LoC (Table 1). Though patients demonstrated a mixed internal and external LoC orientation, responses were skewed towards externality. Chi square test results are shown in Table 2.

**Table 2:** Comparing LoC with sex and educational level

LoC	Sex		Educational level	
	Male (%)	Female (%)	Higher (%)	Lower (%)
High internal	13.5	34.8	25.0	23.3
Low internal	23.8	28.0	27.5	24.3
Chi square	X^2^= 12.9574, *p* = 0.0003		X^2^= 4.5526, *p* = 0.0329	
High external	13.5	32.8	21.5	24.8
Low external	23.8	30.0	31.0	22.8
Chi square	X^2^= 8.9369, *p* = 0.0028		X^2^= 0.0273, *p* = 0.8687	

Sex was significantly associated with internal LoC (X2= 12.9574, p = 0.0003) and external LoC (X2= 8.9369, p = 0.0028). Education was significantly associated with external LoC (X2= 4.5526, p = 0.0329) but not internal LoC (X2= 0.0273, p = 0.8687). The MMAS scores showed that majority (93.3%) of hypertensive patients poorly adhered to their medications (Table 1). Participants cut back or stopped taking their medication without telling the doctor because they felt worse when they took it, or stopped taking the medicine when they felt their blood pressure was under control. About 60% of patients experienced moderate to severe side effects associated with anti-hypertensive medications (Table 1). These side effects included difficulty sleeping, erectile dysfunction, reduced sexual drive, constipation, chest pain, depressed mood, headaches, cough, fatigue, and dizziness.

The logistic regression model results are presented in Table 3. Participants with high internal LoC had 68% lower odds of non-adherence than those with low internal LoC (OR = 0.32 (95% CI 0.11 – 0.95), p = 0.039). Likewise, participants exhibiting low internal LoC had 5.64 times greater odds of being non-adherent than participants with low external LoC (OR = 5.64 (1.14 – 27.87), p = 0.034). Participants scoring low on the “others” subscale of external LoC, had 81% lower odds of non-adherence than their counterparts with high “others” (OR = 0.19 (0.06 – 0.57), p = 0.003) Additionally, participants who experienced moderate to high side effects were more likely to be non-adherent than those who had low experiences of side effects (OR = 4.84 (1.07 – 21.85), p = 0.04). Participants reporting both medication side effects and external LoC had 2.4 times greater odds of non-adherence than participants with side effects and internal LoC (OR = 2.4 (0.35 – 16.31), p <0.001).

**Table 3:** Logistic regression model for LoC and side effects in relation to medication non-adherence behaviour

Variable	Odds ratio	95% confidence interval	*p* value
High Internal LoC (Ref: Low internal Loc)	0.32	0.11 – 0.95	0.039
Low external LoC (Ref: High external LoC)	0.64	0.20 – 2.11	0.468
Low internal LoC/(Ref: Low external LoC)	5.64	1.14 – 27.87	0.034
Low external “others”(Ref: high “others”)	0.19	0.06 – 0.57	0.003
Low external “doctors”(Ref: high “doctors”)	0.64	0.27 – 1.50	0.304
Low external “chance”(Ref: high “chance”)	1.61	0.66 – 3.92	0.296
Moderate to high side effects (Ref: low side effects	4.84	1.07 – 1.85	0.04
Side effects and external Loc (Ref: side effects and internal Loc)	2.4	0.35 – 16.31	<0.001

*p* < 0.05

## Discussion

This study examined the association between personality characteristics (LoC) and medication adherence behaviour while taking into account medication side effects. We found that a very high proportion of patients (93%) did not adhere to their medications. The low level of adherence is similar to that reported by Buabeng and colleagues among hypertensive patients in Ghana [13]. These results underscore the need for interventions to improve adherence levels..

The health LoC construct has been one of the most widely considered predictors of health-related behaviour of patients. Previous research on medication adherence provides evidence linking internal LoC with adherence [20] and non-adherence [31]. The HLoC results from this study showed that hypertensive patients exhibited a blend of LoC. However, it is worth noting that though patients responded highly for both internal and external orientations, the pattern was skewed towards externality particularly, “doctors”. It is possible that although patients feel that they are responsible for their health they also attribute health-related outcomes to doctors. This leads to the belief that the health of hypertensive patients is controllable, either by themselves or doctors, and is not as a result of chance, fate or luck [25]. Patients who are internally-driven have a sense of self-responsibility in following recommendations made by health professionals. According to Wallston and Wallston [25], mixed LoC could be a helpful coping mechanism for patients with chronic conditions and successful management of hypertension may mean patients encouraging themselves to adhere to their doctors’ recommended regimen. In general, there is a paucity of information on the relationship between bilocal LoC and medication adherence behaviour although the general evidence from other studies predominantly associate bilocal LoC with positive attributes and behaviours [43, 44].

The presence of characteristics associated with low internal LoC and high external “others’ accounted for the non-adherence observed. That is, patients with low internal LoC are less likely to take full responsibility for their illness and health behaviours. Additionally, having a high external LoC makes patients more likely to attribute their health actions to external forces instead of taking personal responsibility. Therefore observing a significant relationship between the variables and non-adherence confirmed that patients had a reduced tendency to adhere to prescribed treatment regimen, because they believe they cannot affect their own health behaviours. These findings are similar to studies by Omeje and Nebo (2011) [20] and Combes and Feral (2011) [22].

Though only marginally significant, results showed that women were more inclined towards internal LoC than males. This result contradicts the belief that females are less likely to possess internal LoC than males [45, 46] while corroborating findings by Hamedoglu et al, 2012 [47] as well as Sariçam et al [48]. Internality was also associated with higher education.

Medication side effects were observed to be a potential factor that could repress adherence. The majority of hypertensive patients pointed out that they experienced moderate to high side effects which included difficulty sleeping, erectile dysfunction, reduced sexual drive, constipation, chest pain, depressed mood, headaches, cough, fatigue and dizziness. This corroborates well-documented evidence on medication side effects being a major cause of non-adherence to therapy [12, 14]. External LoC did not considerably influence medication adherence yet when employed, external LoC significantly moderated the relationship between side effects and adherence. The study shows that, external LoC on its own may not be a true predictor of adherence behaviour, but, in the presence of barriers to medication intake such as side effects, the role of external LoC is highly important, contrary to what has been reported in a prior study [49]. However, the possibility of patients managing high external LoC and employing an enhanced internality as an underlining preserve in managing experiences of side effects associated with medications can still be suggested. With the existence of barriers to medication intake, intervention programmes for non-adherence can be directed towards a greater involvement of personality characteristics such as LoC.

Some limitations must be acknowledged. First, although we selected participants from tertiary institutions that serve a diverse population, results may not be widely generalizeable. Second, we included patients with co-morbid health conditions, which might have affected the outcomes we observed.

## Conclusion

Medication non-adherence dominates as a major health problem. The results of this study bring to bear a persistent problem of non-adherence to anti-hypertensive medication among the Ghanaian population, hence, highlighting the need for intervention programmes to enhance adherence to pharmacotherapy. The LoC construct has been one of the most widely considered predictors of health-related behaviour among patients. This study showed that hypertensive patients exhibited a mixed LoC referred to as “bilocal” LoC. Yet this mixed orientation was skewed towards externality. Experiences of medication side effects and medication non-adherence behaviour appear to be linked. Multifaceted intervention programmes that take into account factors like LoC may improve anti-hypertensive medication adherence.
